# Multimorbidity patterns and associated factors in a megacity: a cross-sectional study

**DOI:** 10.11606/s1518-8787.2024058006058

**Published:** 2024-07-10

**Authors:** Ricardo Goes de Aguiar, Daniela Simões, Shamyr Sulyvan Castro, Moises Goldbaum, Chester Luiz Galvão Cesar, Raquel Lucas

**Affiliations:** I Universidade Federal de Alfenas Instituto de Ciências da Motricidade Alfenas MG Brasil Universidade Federal de Alfenas. Instituto de Ciências da Motricidade. Alfenas, MG, Brasil; II Universidade de São Paulo Faculdade de Saúde Pública São Paulo SP Brasil Universidade de São Paulo. Faculdade de Saúde Pública. São Paulo, SP, Brasil; III Universidade do Porto Instituto de Saúde Pública Unidade de Investigação em Epidemiologia Porto Portugal Universidade do Porto. Instituto de Saúde Pública. Unidade de Investigação em Epidemiologia. Porto, Portugal; IV Escola Superior de Saúde de Santa Maria Porto Portugal Escola Superior de Saúde de Santa Maria. Porto, Portugal; V Universidade Federal do Ceará Departamento de Fisioterapia. Fortaleza CE Brasil Universidade Federal do Ceará. Departamento de Fisioterapia. Fortaleza, CE, Brasil; VI Universidade de São Paulo Faculdade de Medicina Departamento de Medicina Preventiva São Paulo SP Brasil Universidade de São Paulo. Faculdade de Medicina. Departamento de Medicina Preventiva. São Paulo, SP, Brasil

**Keywords:** Multimorbidity, Comorbidity, Latent Class Analysis

## Abstract

**OBJECTIVE:**

To identify empirical patterns of multimorbidity and quantify their associations with socioeconomic, behavioral characteristics, and health outcomes in the megacity of São Paulo.

**METHODS:**

This was a cross-sectional study conducted through household interviews with residents aged 20 years or older in urban areas (n = 3,184). Latent class analysis was used to identify patterns among the co-existence of 22 health conditions. Age-adjusted prevalence ratios were estimated using Poisson regression.

**RESULTS:**

The analysis of latent classes showed 4 patterns of multimorbidity, whereas 58.6% of individuals were classified in the low disease probability group, followed by participants presenting cardiovascular conditions (15.9%), respiratory conditions (12.8%), and rheumatic, musculoskeletal, and emotional conditions (12.8%). Older individuals, with lower schooling and lower household income, presented higher multimorbidity prevalence in cardiovascular, respiratory, rheumatic, musculoskeletal, and emotional conditions patterns compared with the low disease probability pattern.

**CONCLUSION:**

The results showed four distinct patterns of multimorbidity in the megacity population, and these patterns are clinically recognizable and theoretically plausible. The identification of trends between patterns would make it feasible to estimate the magnitude of the challenge for the organization of health care policies.

## INTRODUCTION

Despite the number of available studies involving isolated health conditions, there is still a lack of research on multimorbidity. Studies reveal an association between high mortality rates, disabilities and diseases, and the utilization of health care services by citizens, as well as low self-assessment of health status^1,2^. A higher awareness of the combination of conditions affecting individuals is needed to identify the outcomes impacting their health. This knowledge is necessary for setting individual treatment plans and for proposing health care policies and services that consider the fact that different factor combinations can lead to different adverse effects and, consequently, diverse health care needs^3,4^.

The methodology adopted in studies may vary widely. No patterns can be found concerning 1) the definition of multimorbidity, 2) the number of health conditions included, 3) place and adopted methods of data collection, 4) sample selection and size. This fact poses a challenge for those who intend to compare distinct locations and periods^5^. Regarding the definition of multimorbidity, studies have shown great acceptance of the criterion of simultaneous occurrence of two or more health conditions^4^. Nevertheless, a broader definition and analysis is sought to go beyond the simple listing of diseases to one that comprises the complexity of multimorbidity. The European General Practitioners Research Network defined multimorbidity as any combination of a chronic condition with at least one other (acute or chronic) condition, biopsychosocial risk, or risk factor^10^. The prevalence of multimorbidity can vary significantly (2.7% to 95.6%) because of various factors, including age, the number of health conditions, settings (community, primary care, hospital), data source (self-report, database), and geographical factors^11^.

Most of the strategies used by researchers on this topic are restricted to grouping diseases and identifying the presence or absence of multimorbidity by focusing on the list of diagnoses for everyone separately. Another scientific strand utilizes weighted indexes, a limited practice because it demands access to detailed clinical data about health conditions and does not establish patterns of disease aggregation in individuals, which is essential for accurate risk classifications^3,12^. Therefore, a pragmatic approach to describe the multimorbidity construct involves defining patterns of simultaneously occurring health conditions. This can be achieved through methods such as Latent Class Analysis (LCA), which is based on a probabilistic model in which, from the responses to different collected variables, similarities among individuals are sought, generating a new non-collected (or latent) variable, which allows the identification and classification of individuals into smaller numbers of distinct clusters. This approach is centered on individuals and makes their organization into subgroups feasible, which present similar patterns of responses and/or conditions^3,13,14^.

Considering aspects such as 1) population aging, 2) the increased prevalence of individuals presenting chronic health conditions and living in uneven socioeconomic realities, and 3) difficulties individuals face in accessing health care facilities, we sought to identify empirical patterns of multimorbidity and quantify their associations with socioeconomic, behavioral traits, and health outcomes in the megacity of São Paulo.

## METHODS

This study used data from the 2015 Health Survey from the city of São Paulo (ISA-Capital), a cross-sectional population-based study consisting of household interviews, in which probabilistic sample-based methods were used by conglomerates in two stages: census tracts and households, weighted to compensate for different selection probabilities. The sample was divided into four domains by age and sex, and 150 census tracts were drawn. Household sets of sufficient size to reach the number of interviews for each domain were randomly selected, and all individuals who met the indicated domain were interviewed directly.

Individuals aged 20 and above living in permanent private residences in the urban area of São Paulo were selected, totaling 3,184 participants, of whom 152 were excluded due to loss in at least one of the conditions considered, using n = 3,032. A sample weight was associated with each of the individuals, which was calculated according to three components: 1) Design weight, which takes into account the sampling fractions of the two draw stages; 2) Nonresponse adjustment, which takes response rates into account; 3) Post-stratification, which adjusts the sample distribution by gender, age group, and region of the household, according to the distribution of the population in the city. The sample calculation was performed using the following parameters: estimated proportion of 0.50; sampling error of 0.10; a 95% confidence level; and a design effect of 1.5. The following algebraic expression was used to estimate the minimum sample size to estimate proportions under complex samples: $n=\frac{P_{*}(1-P)}{{\left(\frac{d}{2}\right)}^{2}}*deff$ , where *n* is the sample size, *P* is the parameter to be estimated, z = 1.96 is the value in the reduced normal curve related to the 95% confidence level of the confidence intervals, *d* is the sampling error, and *deff* is the effect of the design^15^.

Regarding the health conditions to be considered, the literature suggests the use of at least 12 conditions, although there is no consensus on which ones should be included^5^. All the 22 variables present in the survey were included in the study: 1) high blood pressure; 2) diabetes; 3) angina; 4) heart attack; 5) cardiac arrhythmia; 6) other heart diseases; 7) cancer; 8) arthritis, rheumatism, or arthrosis; 9) osteoporosis; 10) asthma or asthmatic bronchitis; 11) emphysema, chronic bronchitis or chronic obstructive pulmonary disease (COPD); 12) rhinitis; 13) chronic sinusitis; 14) other lung diseases; 15) tendonitis, repetitive strain injury (RSI) or work-related musculoskeletal disorder (WMSD); 16) lower limbs varicose veins; 17) stroke; 18) other vascular, arterial or circulatory disease; 19) high cholesterol; 20) spine conditions or disorders; 21) emotional or mental disorder, such as anxiety, depression, panic disorder, obsessive-compulsive disorder (OCD), schizophrenia or alike; and 22) other chronic diseases, in addition to the above mentioned. For each of them, the respondent had to answer the following question “Has any doctor ever informed you that you suffer from ...” followed by the condition.

For the characterization of individuals, sociodemographic variables were used: sex (female, male), age group (20 to 29, 30 to 39, 40 to 49, 50 to 59, 60 to 69, ≥70), race/skin color (white, brown, black, other), marital status (with a partner, no partner), and schooling (preschool, elementary, high school or technical school, unfinished higher education, higher education and/or postgraduate studies). The anthropometric variable was the Body Mass Index (underweight, normal, overweight, obesity). Health-related behaviors were considered: smoking (yes, no) and physical activity ≥150 min/week (yes, no). There were also economic variables: current employment status (employed, unemployed) and median household income (1^st^, 2^nd^, 3^rd^, 4^th^ quartiles). Self-reported morbidity and disabilities were collected: presence of common mental disorders using the Self-Reporting Questionnaire (SRQ-20) (yes, no), health status (excellent/very good, good, normal, bad/very bad), functional or activity limitation (yes, no) and bedridden (yes, no), along with utilization of health care facilities: last visit to a health care unit (less than 2 weeks, 15 to 30 days, 1 to 3 months before, 3 to 6 months, 6 to 12 months, more than one year), and hospitalizations or surgeries (yes, no). All variables were selected as independent.

Regarding data analysis, prevalence estimates and confidence intervals (95%) were retrieved from each independent variable. To analyze the outcome, we employed LCA using self-reported health conditions. LCA enabled the identification of distinct multimorbidity patterns among participants with the lowest Bayesian Information Criterion (BIC). The resultant variable represented the classification of individuals into specific multimorbidity patterns and served as the dependent variable.

Poisson regression was employed for the association analysis adjusted for sample weight and aspects related to the complex sample design to ensure the robustness of our analyses, considering the sample structure. Statistical software R version 4.0.3, poLCA, epitools, and lmtest packages were used.

## RESULTS

Table 1 summarizes the characteristics of the participants. The majority comprised women (53.5%), and 24.2% were aged between 30 and 39. Just over half reported themselves as having a white race/skin color, 59.0% declared living with a partner, and 19.4% had finished, at least, higher education. Regarding body mass index, 41.8% presented with normal weight, most (81.3%) met the WHO recommendation of 150 minutes of global physical activity per week, and about two-thirds had never smoked. At the time of data collection, 65.6% of participants were employed and 29.1% were classified in the 4th quartile of the median household income report. In addition, 51.9% self-evaluated their health status as good, 80.6% had not presented any common mental disorder, and 61.0% had not been limited in their functional/activity. Fourteen percent reported not having utilized health care services, and 8.4% reported hospitalizations or surgeries within the previous year.

**Table 1:** Sociodemographic, anthropometric, and lifestyle characteristics and health events of the participants.

Characteristics	n (3032)	% (100.00)	95%CI
Sex			
Female	1753	53.46	51.43–55.48
Male	1279	46.54	44.52–48.57
Age range			
20–29	549	22.7	20.97–24.52
30–39	597	24.23	22.47–26.09
40–49	499	19.49	17.89–21.19
50–59	445	15.71	14.31–17.23
60–69	530	10.29	9.38–11.28
≥70	412	7.58	6.81–8.43
Race/skin color			
White	1560	52.35	50.33–54.36
Brown	974	32.16	30.33–34.05
Black	313	10.28	9.15–11.54
Other	168	5.2	4.39–6.16
Marital status			
With a partner	1738	59.04	57.05–61.01
With no partner	1287	40.96	38.99–42.95
Schooling			
Preschool (0–4 y.o.)	513	11.85	10.77–13.02
Elementary (5–9 y.o.)	800	22.76	21.24–24.36
High school or technical school	1031	36.56	34.64–38.53
Unfinished Higher education	222	9.41	8.19–10.79
Higher education and/or Postgraduate studies	451	19.41	17.67–21.27
Body mass index (BMI)			
Underweight	239	5.75	4.93–6.69
Normal	1241	41.76	39.76–43.78
Overweight	864	31.97	30.09–33.91
Obesity	625	20.52	18.92–22.21
WHO recommended physical activity (≥150 min/week)			
Yes	2408	81.29	79.7–82.79
No	598	18.71	17.21–20.30
Smoking			
No	2010	67.18	65.27–69.04
Yes	1019	32.82	30.96–34.73
Employment status			
Employed	1767	65.61	63.73–67.44
Unemployed	1257	34.39	32.56–36.27
Median household income			
1st quartile	579	22.74	20.94–24.64
2nd quartile	590	24.25	22.38–26.22
3rd quartile	557	23.87	22.01–25.84
4th quartile	562	29.14	26.93–31.45
Common Mental Disorder (CMD)			
Yes	634	19.41	17.89–21.01
No	2315	80.59	78.99–82.11
Health status			
Excellent/very good	568	21.0	19.33–22.77
Good	1539	51.9	49.88–53.91
Normal	798	23.7	22.09–25.39
Bad/very bad	122	3.4	2.81–4.12
Health status comparison (≥ 60 y.o.)			
Better than 1 year before	234	23.78	20.96–26.85
Same as 1 year before	505	54.68	51.16–58.15
Worse than 1 year before	200	21.54	18.76–24.61
Functional or Activity Limitations			
Yes	228	39.0	34.57–43.62
No	364	61.0	56.38–65.43
Bedridden			
No	463	78.01	73.83–81.68
Yes	129	21.99	18.32–26.17
Last-time health care service utilization			
Less than 2 weeks	572	18.36	16.84–19.99
15–30 days	449	14.43	13.10–15.87
1–months	711	22.49	20.87–24.20
3–6 months	462	16.00	14.54–17.58
6–12 months	424	14.74	13.35–16.25
More than 1 year	402	13.97	12.63–15.43
Surgeries and hospitalization			
No	2788	91.59	90.31–92.72
Yes	242	8.41	7.28–9.69

95%CI: 95% confidence interval.

Four classes were chosen after considering the analysis of BIC values, pathophysiological plausibility, and discrimination of conditions between patterns. Each class presented the following profiles: pattern 1 (respiratory conditions), in which individuals were more susceptible to rhinitis (65.1%), chronic sinusitis (47.3%), asthma or asthmatic bronchitis and emphysema (24.2%), chronic bronchitis or chronic obstructive pulmonary disease (8.2%); pattern 2 (cardiovascular conditions), consisting of a higher probability of high blood pressure (81.0%), diabetes mellitus (40.8%), heart attack (9.6%), and cerebrovascular accident (8.3%); pattern 3 (rheumatic, musculoskeletal and emotional conditions), characterized by a higher probability of arthritis, rheumatism, and arthrosis (59.9%), spine conditions or disorders (59.0%), high cholesterol (46.8%), and emotional or mental disorder (40.9%); and pattern 4 (low disease probability), comprising individuals presenting a low probability of suffering from any of the 22 selected conditions (Figure).

**Figure:**
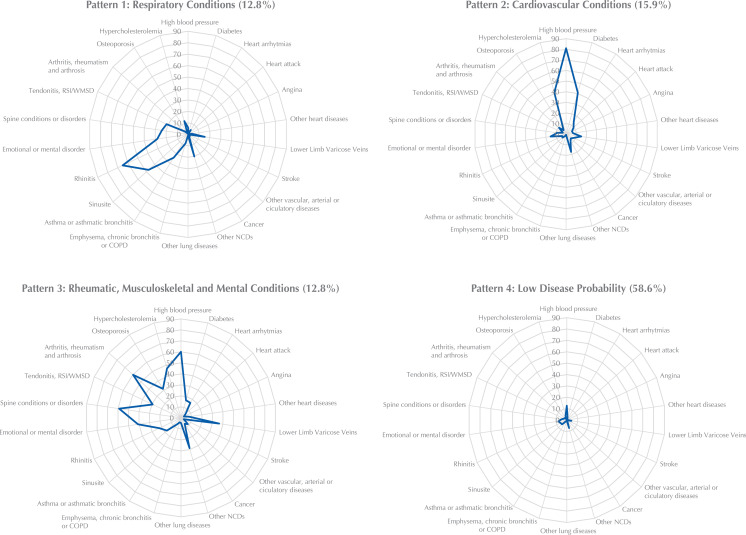
Marginal percentage of subjects suffering from each health condition in each assigned pattern to predict class classification among the population*.

COPD: chronic obstructive pulmonary disease; RSI/WMSD: Repetitive Strain Injury/Work-Related Musculoskeletal Disorders, NCD: non-communicable disease.

Table 2 presents a comparison of prevalences for each pattern of multimorbidity among subgroups, generated by the categories of independent variables. Pattern 4 was set as a reference, and the prevalence ratios between each category of independent variables of the three other multimorbidity patterns were calculated. Notably, age group was a determining prevalence factor, indicating the need for controlling effects by adjusting the Poisson regression model.

**Table 2:** Association between patterns of health conditions and sociodemographic, anthropometric, health-related behaviors, economic status, reported morbidity and disabilities, and utilization of health care services, ISA Capital, São Paulo, 2015.

Variables	Pattern 1	Pattern 2	Pattern 3
		
	Respiratory conditions	Cardiovascular conditions	Rheumatic, muscular/skeletal, and mental conditions
		
	PR (95%CI)	PR (95%CI)	PR (95%CI)
Age group			
20–29	1	1	1
30–39	4.85 (4.85–4.85)	2.91 (2.91–2.91)	1.02 (1.02–1.02)
40–49	15.56 (15.56–15.56)	14.62 (14.62–14.62)	1.26 (1.26–1.26)
50–59	84.3 (84.3–84.3)	47.42 (47.42–47.42)	1.85 (1.85–1.85)
60–69	164.69 (164.69–164.69)	125.77 (125.77–125.77)	2.13 (2.13–2.13)
70+	473.67 (473.67–473.67)	316.05 (316.05–316.05)	4.4 (4.4–4.4)
Gender			
Female	1	1	1
Male	0.46 (0.22–0.96)	1.52 (0.72–3.21)	2.25 (1.59–3.18)
Race/skin color			
White	1	1	1
Black	0.53 (0.28–1)	0.69 (0.37–1.28)	1.21 (0.95–1.53)
Other	1.29 (0.72–2.33)	1.3 (0.66–2.54)	0.89 (0.7–1.14)
Brown	0.54 (0.3–0.99)	0.57 (0.31–1.05)	1.24 (0.99–1.56)
Marital status			
With a partner	1	1	1
With no partner	1.3 (0.55–3.11)	1.34 (0.55–3.26)	0.88 (0.47–1.67)
Schooling			
Preschool (0 to 4 y.o.)	1	1	1
Elementary (5 to 9 y.o.)	0.36 (0.13–0.96)	0.24 (0.09–0.66)	0.55 (0.21–1.44)
High school or technical school	0.09 (0.04–0.19)	0.06 (0.02–0.15)	0.45 (0.22–0.93)
Incomplete higher education	0.05 (0.02–0.15)	0.04 (0.01–0.11)	0.25 (0.12–0.52)
Complete higher education and/or post-graduation	0.12 (0.05–0.33)	0.12 (0.05–0.3)	0.34 (0.14–0.83)
Work status			
Employed	1	1	1
Unemployed	5.69 (2.46–13.16)	4.59 (2.29–9.23)	0.98 (0.67–1.42)
Medium household income			
1st quartile	1	1	1
2nd quartile	0.82 (0.37–1.82)	0.63 (0.31–1.27)	0.81 (0.63–1.06)
3rd quartile	0.61 (0.29–1.26)	0.6 (0.25–1.41)	0.7 (0.49–0.98)
4th quartile	0.69 (0.37–1.28)	0.73 (0.36–1.48)	0.56 (0.45–0.69)
Self-rated health compared with the previous year			
Excellent/very good	1	1	1
Good	2.35 (1–5.52)	1.6 (0.77–3.35)	0.83 (0.59–1.18)
Normal	3.34 (1.34–8.32)	1.96 (0.87–4.41)	0.37 (0.25–0.55)
Bad or very bad	3.5 (1.33–9.25)	2.15 (0.92–5.01)	0.2 (0.11–0.35)
Self-rated health compared to the previous year (>60 y.o.)			
Same as 1 year ago	1	1	1
Better than 1 year ago	0.68 (0.49–0.95)	0.51 (0.38–0.7)	0.4 (0.29–0.55)
Worse than 1 year ago	1.7 (1.05–2.76)	1.37 (0.89–2.1)	0.57 (0.38–0.86)
Functional or Activity Limitations			
No	1	1	1
Yes	0.61 (0.35–1.07)	1.27 (0.67–2.41)	0.69 (0.48–1.01)
Bedridden			
No	1	1	1
Yes	0.85 (0.39–1.85)	1.2 (0.56–2.54)	0.74 (0.41–1.32)
Body mass index (BMI)			
Normal	1	1	1
Underweight	4.29 (2.02–9.11)	4.04 (2.12–7.7)	1.61 (0.7–3.71)
Overweight	0.59 (0.25–1.37)	0.5 (0.24–1.03)	0.88 (0.62–1.24)
Obese	1.95 (1–3.83)	1.69 (0.8–3.6)	1.06 (0.77–1.45)
WHO recommended physical activity (≥ 150 min/week)			
No	1	1	1
Yes	0.37 (0.16–0.87)	0.3 (0.12–0.71)	0.76 (0.55–1.05)
Smoking			
No	1	1	1
Yes	1.65 (0.78–3.49)	1.29 (0.62–2.67)	1.14 (0.81–1.59)
Last-time health care service utilization			
Less than 2 weeks	1	1	1
15–30 days	1.31 (0.71–2.44)	1.29 (0.6–2.78)	1.73 (1.19–2.51)
1–3 months	1.2 (0.65–2.23)	1.03 (0.48–2.23)	1.54 (1.05–2.26)
3–6 months	0.87 (0.47–1.63)	0.72 (0.35–1.48)	1.63 (1.2–2.21)
6–12 months	0.43 (0.23–0.81)	0.55 (0.26–1.14)	1.86 (1.27–2.72)
More than 1 year	0.23 (0.1–0.49)	0.22 (0.09–0.58)	2.87 (2–4.12)
Hospitalizations and/or surgeries			
No	1	1	1
Yes	0.97 (0.47–2.03)	1.21 (0.49–2.98)	0.65 (0.46–0.91)
Emotional health condition			
No	1	1	1
Yes	1.51 (0.64–3.52)	0.65 (0.3–1.4)	0.35 (0.26–0.48)

*PR: Prevalence Ratio; 95%CI: 95% confidence interval; PR adjusted for age.

Pattern 4 (low disease probability) was used as the reference category.

When compared with the pattern of low disease probability, the pattern of respiratory conditions had a statistically greater representativity in women and smaller in self-declared brown skin color individuals. Regarding the pattern of rheumatic, musculoskeletal, and emotional conditions, men were more prevalent. Relating to schooling, a statistically significant association between this variable and almost all the categories, except elementary school individuals in pattern 3, was verified, as multimorbidity showed less incidence among higher schooling individuals.

Underweight individuals had a higher prevalence of multimorbidity in patterns 1 and 2. The highest prevalence of multimorbidity was also associated with obesity in pattern 1. Patterns 1 and 2 were statistically associated with the level of global physical activity, comprising individuals who met the WHO recommendations, thus showing a lower incidence of multimorbidity. Smoking did not set statistically significant associations.

Concerning variables related to the economic status of individuals, a statistically significant association was identified among unemployed individuals within the respiratory and cardiovascular patterns. Regarding the musculoskeletal pattern, an association was identified between multimorbidity and higher household income (3rd and 4th quartiles).

Regarding self-reported morbidity and disabilities, in patterns 1 and 3, a statistically significant association was identified for health status self-assessment at the time of data collection. For individuals aged 60 years and older, there was a statistically significant association when compared with their health status in the previous year, in all patterns.

Statistically significant associations were identified on the prevalence of the last time utilization of health care services. In pattern 1, individuals who reported having utilized the services between 6 and 12 months before and more than 1 year before the survey presented lower prevalences of multimorbidity. The same occurred to those who utilized health care services for the last time more than 1 year before, among individuals in pattern 2. In pattern 3, however, there was an inversion, revealing a higher prevalence among individuals who utilized the services longer before. In this pattern, an association with hospitalizations or surgeries was also observed.

## DISCUSSION

The designs of previously available studies were heterogeneous because of aspects such as the adopted concept of multimorbidity, number and types of conditions included, source of data collection, and methods of analysis^6,16,17^. Thus, we sought to define categories of multimorbidity based on natural patterns of population clustering. The adopted approach is believed to result in more generalizable outcomes, given the use of a well-accepted sample, applied to studies on various conditions for more than a decade, and quantitatively included health conditions and compatible characteristics with other studies^6,8,12,16^. This analysis produced realistic, clinically recognizable, and theoretically plausible aggregation patterns in the affected individuals.

The pattern of those with a low disease probability corresponded to almost 60% of the participants, followed by the patterns of cardiovascular conditions (15.9%), respiratory conditions (12.8%), and rheumatic, musculoskeletal, and emotional conditions (12.8%). Other studies on patterns of multimorbidity concluded that the class presenting low disease probability comprised more than half of the individuals (54.1% in the United States of America^19^ and 68.4% in Portugal^17^). A study in Brazil identified higher proportions of metabolic and musculoskeletal conditions, followed by mental and respiratory conditions^20^. Regarding cardiovascular conditions, other studies have reached similar results, adopting a pattern that concentrated conditions such as high blood pressure, diabetes, and heart disease, resulting from metabolic risk factors inherent to the current lifestyle, thus raising the probability for the development of those conditions^1,17,21^.

All the studied patterns presented a statistically significant association with aging, as described in the literature^7,20,22,23^, emphasizing respiratory and cardiovascular patterns, and less intensely present in rheumatic, musculoskeletal, and emotional patterns. Regarding the pattern of respiratory conditions, some of the conditions may be attributed to the air quality in a megacity such as São Paulo. Smoking, which might be another associable factor^17^, despite presenting a higher prevalence ratio compared with other patterns, was not statistically associated with any of them.

The association of rheumatic, musculoskeletal, and emotional conditions is consistent with studies^9,17^. Although it was expected that the presence of multimorbidity in individuals would lead to greater contact with health care services and, consequently, a better quality of medical care for these individuals, this does not seem to be the case. Some conditions are undertreated, especially when they involve emotional or mental disorders. Health care services are fragmented, and individuals with multimorbidities become more susceptible to medical errors^23^. A systematic review pointed to a greater risk to the safety of individuals with mental disorders associated with physical conditions due to errors in prescription or use of medication, non-adherence to treatments, adverse events caused by drugs or interventions, among others^24,25^.

Concerning gender, respiratory patterns presented a higher prevalence among women, whereas in rheumatic, musculoskeletal, and emotional patterns, the prevalence was higher among men. Although this relationship has not been fully established, most studies point to a higher prevalence among women. A systematic review identified a significantly higher prevalence of women in 64.3% of the studies^7^. An explanation for the higher presence of multimorbidity among women in studies is the fact that women seek health care services more often, so that they are diagnosed^26^. The resistance of men, particularly older individuals, to seeking healthcare^27^ highlights the higher prevalence of multimorbidity among women.

Regarding race/skin color, a national study did not identify differences between groups^20^. In São Paulo, although black and brown people presented a lower prevalence of multimorbidity within the respiratory and cardiovascular patterns, a situation that may be associated with their difficulties in accessing health care facilities, a statistically significant difference was only identified for those who self-declared to be brown within the respiratory pattern. Black and brown populations in Brazil have, as a rule, lower schooling, a variable that can be used as a proxy for income. In this study, individuals with higher schooling showed lower prevalence in almost all patterns and schooling. Except for elementary school within the rheumatic, musculoskeletal, and emotional patterns. Findings from other studies are similar both in terms of schooling^20,25^ and income, showing a higher prevalence of multimorbidity in groups living in lower socioeconomic classes, reaching a two- to three-fold higher incidence among poorer individuals^7,22,28^.

Participants with respiratory and cardiovascular patterns who reported being unemployed had a higher prevalence of multimorbidity. A study drew attention to the high prevalence of multimorbidity among individuals in economically active age groups and the consequences of this phenomenon for the productive system. It pointed out as a hypothesis for the development of concomitant conditions, the submission of workers to various work-related risks, and highlighted the difficulties faced by these individuals to access health care facilities, which usually operate in working-hour shifts, at the same periods these individuals are at work^20^.

Those classified in the pattern of respiratory conditions who reported normal, bad, or very bad health status had higher statistically significant multimorbidity prevalence. The opposite was observed among individuals with rheumatic, musculoskeletal, and emotional patterns, with lower prevalence among those who reported average, bad, or very bad health status. For the elderly population, when comparing their health status at the time of the survey with the previous year, a statistically significant association of those who stated that their health status improved throughout the year was observed, with a lower prevalence of multimorbidity for all the patterns. There is evidence in the literature concerning the decline in the quality of life for individuals living with multimorbidity, and anachronistically, the burden of health conditions among individuals under 65 years of age is higher^23,29^.

Studies have shown an association between multimorbidity and a more frequent utilization of health care services, as well as higher costs for individuals and health care systems^17,30^. The results of the present study were not sufficient to point in the same direction. This may have occurred because of the peculiarities of the organization of health care services in Brazil. Although the country has an established universal health system (SUS), which presents the integrality of health care among its principles, it still faces challenges in ensuring adequate health care to meet the population’s needs. The same challenge is posed when focusing on individuals living with multimorbidity, even in a city such as São Paulo, which is a center for training and health service provision. However, the city health care network comprises a high number of specialists and an approach focused on isolated diseases, especially the prevalent ones, such as high blood pressure and diabetes^12^. In addition to the difficulties concerning access to health care facilities in a megacity, the long distances to be covered to reach the health care facilities, the opening hours in periods when the individuals are usually working, and the long waiting lines, among other factors, inhibit the demand for health care services. As the population ages and the prevalence of multimorbidity increases, policies must rely on multidisciplinary teams working interprofessionally. Social health policies must take a comprehensive look at individuals, not just diseases, acting toward the promotion of health and disease prevention, avoiding functional/activity limitations, observing the interactions between health conditions, seeking adequate attention to needs at an affordable cost for individuals and the health care system, and promoting, therefore, better quality of life for all.

The present study is limited by the fact that the diagnosis of health conditions was self-reported, which might have led to underreporting due to memory biases, and because certain conditions do not require the individual to seek health care services. Although concerning musculoskeletal conditions, it has been shown that self-reporting in population surveys is better for estimating the prevalence of multimorbidity than routine clinical information^7^, another limitation relies on considering only the diagnosis of the conditions, not considering their severity and length. Such a situation probably impacts the clinical life and evolution of individuals’ health status. In addition, the study excluded individuals who lived in long-term care facilities or who were hospitalized at the time of the survey. Besides, the inefficiency of the results in inferring causality and the processes that led to the grouping of conditions must be considered. Also, no additional analyses were performed to identify errors in the classification of individuals into classes, such as normal weight, which should be conducted in further studies.

This study is innovative in applying an empirical model-based approach to identify patterns of multimorbidity in a megacity. Four distinct patterns of co-occurrence of health conditions in the city population were identified, and these patterns are clinically recognizable and theoretically plausible. We considered the guidelines of conceptual models and systematic reviews to define the criteria for the selection of health conditions and outcome variables^4^. LCA has well-described procedures and is widely used in the literature, allowing the estimation of the probability for each individual belonging to a certain class.

Studies on the prevalence of multimorbidity are essential to estimate the magnitude of the problem and enable the organization of health care policies. Thus, managers and professionals must have access to disaggregated data that will allow planning of the health care network and management of the medical care of individuals living with multimorbidity. The impacts of a treatment on other health conditions cannot be ignored, as there is a possibility that some of them may be masked by multiple overlapping symptoms. This urges health systems to be ready to serve these individuals through an interprofessional and person-centered approach.
